# Antifungal Volatile Organic Compounds from *Talaromyces purpureogenus* CEF642^N^: Insights from One Strain Many Compounds (OSMAC) Strategy for Controlling *Verticillium dahliae* in Cotton

**DOI:** 10.3390/jof11050332

**Published:** 2025-04-22

**Authors:** Peng Li, Yalin Zhang, Hongjie Feng, Jinglong Zhou, Lihong Zhao, Heqin Zhu, Feng Wei, Zili Feng

**Affiliations:** 1 State Key Laboratory of Cotton Bio-Breeding and Integrated Utilization, Institute of Cotton Research, Chinese Academy of Agricultural Sciences, Anyang 455000, China; peng_li0429@163.com (P.L.); yalinzhang2012@163.com (Y.Z.); fenghongjie@caas.cn (H.F.); zhoujinglong@caas.cn (J.Z.); zhaolihongqq@163.com (L.Z.); heqinanyang@163.com (H.Z.); 2 Western Agricultural Research Center, Chinese Academy of Agricultural Sciences, Changji 831100, China

**Keywords:** *Talaromyces purpureogenus* CEF642^N^, *Verticillium dahliae*, volatile organic compounds, antifungal activity, transcriptome analysis

## Abstract

Verticillium wilt is a devastating soil-borne disease that significantly impacts cotton production, necessitating the development the effective biofumigants for its control. In this study, the inhibitory effect of total volatile organic compounds (VOCs) produced by *Talaromyces purpureogenus* CEF642^N^ against *Verticillium dahliae* were evaluated using the one strain many compounds (OSMAC) strategy and analyzed through volatile metabolome. CEF642^N^ was found to produce two primary VOCs, 3-octanol and 2-octenal, (E)-, both of which demonstrated significant antifungal activity. Transcriptome analysis of mycelium grown on various solid media revealed notable differences in the expression of genes associated with arachidonic acid metabolism, lipoxygenase (LOX), and lytic enzymes. These findings provide a foundation for future research aimed at identifying key genes involved in the eight-carbon volatile biosynthetic pathway.

## 1. Introduction

*Verticillium dahliae* is a soil-borne hemibiotrophic fungus responsible for causing vascular disease in plants, leading to significant economic losses worldwide. This pathogen infects over 400 dicotyledon plant species, including annual herbs, perennials, and woody plants. In cotton crops, Verticillium wilt alone results in an average yield reduction of 10–35% [1]. Plant diseases, in general, contribute to substantial global crop losses, adversely affecting both the quality and quantity of agricultural produce. In response to the global shift toward reducing chemically synthesized pesticides, plant disease management has increasingly relied on biocontrol technologies, strategies, and approaches. Fungal antagonists, in particular, play a critical role in controlling plant pathogens and are widely utilized as biocontrol agents [2]. Fungal volatile organic compounds (VOCs) have emerged as a promising alternative to chemical pesticides, offering potential benefits for crop protection and overall plant health.

The application of fungal VOCs as pesticide alternatives has garnered considerable attention [3,4]. For instance, VOCs produced by *Trichoderma atroviride* inhibit the growth of *Phytophthora infestans* and induce significant morphological and ultrastructural damage to its mycelium, including cell deformation, collapse, and organelle destruction [5]. Similarly, 6-pentyl-2H-pyran-2-one, a VOC from *Trichoderma erinaceum*, strongly suppresses the growth and sporangial germination of *Peronophythora litchii*, causing severe intracellular damage [6]. Citronellol, produced by *Kluyveromyces marxianus* YG-4, inhibits *Penicillium expansum* by damaging cell structures, disrupting redox system, reducing antioxidant enzymes activity, and inducing oxidative damage [7].

These VOCs, which function as antimicrobial “weapon systems” for fungi, are produced under specific environmental conditions or during particular stages of the fungal life cycle. However, replicating these complex conditions in laboratory settings remains challenging, thereby limiting our ability to exploit the chemical diversity of fungi. Consequently, only a fraction of fungal secondary metabolites (SMs) has been characterized, leaving significant potential for the discovery of novel bioactive compounds [8,9]. The one strain many compounds (OSMAC) approach, which involves systematically altering culture conditions, has proven effective in exploring microbial SMs diversity [10]. This strategy can activate silent gene clusters in microorganisms, leading to the production of additional SMs. Due to its simplicity and efficacy, the OSMAC approach has been widely adopted [11,12].

Advances in genetics and bioinformatics have further enhanced natural product discovery. Integrating genomic and transcriptomic technologies provides a practical and efficient strategy to overcome the limitations of traditional methods. For instance, comparative transcriptome analysis can inform the optimization of fungal culture conditions to improve the yield of target compounds [13].

In this study, the OSMAC strategy was employed to investigate the VOCs produced by *Talaromyces purpureogenus* CEF642^N^ across three different media. The VOCs exhibited varying inhibition rates against *V. dahliae*, and the underlying reasons for these differences were explored through comparative analyses of their volatile metabolomes. Two major VOCs demonstrated significant inhibitory effects on hyphae growth and spore germination. Additionally, comparative transcriptome analyses were conducted to identify potential genes involved in their biosynthesis, providing a foundation for future research into the production and application of these compounds.

## 2. Materials and Methods

### 2.1. Strains and Culture Conditions

The antagonist strain CEF642^N^, used in this study, is an endophytic fungus isolated from healthy cotton roots and identified as *Talaromyces purpureogenus* through morphology analysis and ITS sequencing [14]. The pathogenic strain *Verticillium dahliae* (accession number Vd076) was isolated from cotton plants exhibiting Verticillium wilt and was identified as a pathogenic strain [15]. The compositions of the various media used in this study are provided in Supplementary Materials Table S1.

### 2.2. Inhibitory Activity of Total VOCs from Different Media

The antagonistic effect of VOCs produced by CEF642^N^ on *V. dahliae* was evaluated using a double Petri dish assay [16]. In the treatment group, one Petri dish was inoculated with a 5 mm plug of the antagonistic fungus (CEF642^N^), while the other contained a 5 mm plug of the pathogenic fungus (*V. dahliae*). Both dishes were filled with the same medium, then docked face-to-face with CEF642^N^ oriented upward and *V. dahliae* oriented downward, followed by parafilm sealing for co-cultivation. In the control group, only the pathogenic fungus was inoculated and cultivated independently. All cultures were incubated at a constant temperature of 25 °C for 15 days. Colony diameters were measured and photographed every 3 days, with each experiment repeated five times. The inhibition rate was calculated using the following formula:

$$Inhibition\;rate\;(\%)=\frac{\left(Cd-Td\right)}{Cd}\times 100$$

where Cd represents the colony diameter of the control group minus 5 mm, and Td represents the colony diameter of the treatment group minus 5 mm.

### 2.3. Identification of VOCs Produced by CEF642^N^ from Different Media

Strain CEF642^N^ was cultivated on Czapek agar (CA), glucose yeast extract soluble starch agar (GYESA), and yeast extract peptone dextrose agar (YPDA) media at 25 °C for 15 days. Mycelium was scraped from the medium surface using a sterile scalpel and 150 mg aliquots were transferred into 20 mL headspace (HS) vials containing 10 μL of 2-octanol as the internal standard for relative VOC quantification.

SPME conditions: The PAL (Prep And Load) rail system was programmed with the following solid-phase microextraction (SPME) parameters: incubation temperature of 60 °C, consisting of 15 min preheating, 30 min incubation, and 4 min desorption.

GC-MS configuration: An Agilent 7890 gas chromatograph (GC) coupled to a 5977B mass spectrometer (MS) was employed. Chromatographic separation used a DB-Wax column (30 m × 250 μm × 0.25 μm) with helium carrier gas at 1 mL/min. The oven temperature program initiated at 40 °C (hold 4 min), ramped at 5 °C/min to 245 °C (hold 5 min). Electron Impact (EI) ionization was performed at −70 eV. Compound identification utilized the NIST library [14].

### 2.4. In Vitro Antifungal Activity Assay

To evaluate the antifungal activity of VOCs differentially produced in GYESA and YPDA media, we conducted mycelial growth inhibition assays using commercial standard compounds: 3-octanol and 2-octenal, (E)-. The experimental setup was adapted from the confrontation culture method with modifications [17]. In place of the direct application of biocontrol fungus, we positioned Oxford cups containing graded volumes of VOCs on potato dextrose agar (PDA)-containing Petri dishes. Each dish (total volume 70 mL) contained 20 mL of PDA medium. VOC concentrations were standardized according to the 50 mL headspace volume. All sealed dishes were incubated at 25 °C for 15 days, followed by the calculation of mycelial growth inhibition rates as outlined in Section 2.2.

The inhibition rate of *V. dahliae* spore germination was determined using a modified method [18]. A 1 μL dose of VOCs was applied to a spore suspension with a concentration of 1 × 10^6^ spores/mL. The spore germination rate was calculated as the number of germinated spores divided by the total number of spores in the same field of view, with germination defined as the germ tube length exceeding half the spore diameter. The inhibition rate was computed as follows:

$$Inhibition\;rate\;(\%)=\frac{\left(Cg-Tg\right)}{Cg}\times 100$$

where Cg represents the germination rate of the control group, and Tg represents the germination rate of the treatment group.

### 2.5. Transcriptome Analysis of Different Solid Media

CEF642^N^ was inoculated onto various solid media and allowed to grow for 15 days. The mycelium was then scraped using a scalpel, flash-frozen in liquid nitrogen, and sent to Personal Biotechnology Co., Ltd. (Shanghai, China) for transcriptome sequencing. For the GYESA and YPDA media, one replicate was prepared for every five dishes, while for the CA medium, one replicate was prepared for every six dishes. Each experiment was repeated three times.

### 2.6. Statistical Analyses

Statistical analyses were performed using SPSS 19.0, employing the least significant difference (LSD) test and one-way analysis of variance (ANOVA) for multiple comparisons. Statistical significance (*p* < 0.05) is denoted in figure legends using distinct lowercase letters. Data are expressed as means ± standard deviation (SD). All experiments were independently repeated at least three times to ensure reproducibility.

## 3. Results

### 3.1. Inhibitory Effects of Total VOCs from Various Solid Media

**Figure 1:**
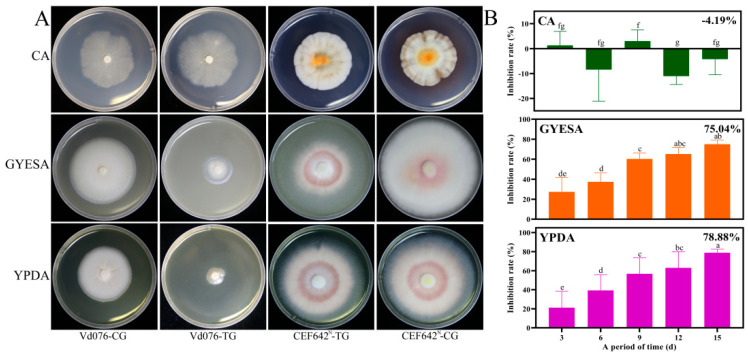
Inhibitory effect of total VOCs produced by CEF642^N^ on *Verticillium dahliae* across three media. (**A**) Photographs of CEF642^N^ and Vd076 at day 15 of double Petri dish assay. (**B**) Time course of Vd076 inhibition rates by total VOCs produced by CEF642^N^ in different media. CG: control group; TG: treatment group.

The total VOCs produced by CEF642^N^ exhibited varying inhibitory effects against Vd076 depending on the culture medium. While CEF642^N^ VOCs showed no inhibitory efficacy against the pathogen when cultured on CA medium (Figure S1), strong inhibitory effects were observed when CEF642^N^ was grown on GYESA and YPDA media (Figures S2 and S3). Specifically, inhibition rates of 75.04% and 78.88% were recorded on the fifteenth day of the double Petri dish assay for the GYESA and YPDA media, respectively (Figure 1). These differences may be attributed to variations in biomass, as well as the types and concentration of VOCs produced. It is hypothesized that CEF642^N^ generates volatiles with potent antifungal activity against *V. dahliae* when cultured on GYESA and YPDA media.

### 3.2. Identification and Comparative of CEF642^N^ VOCs from Different Solid Media

**Figure 2:**
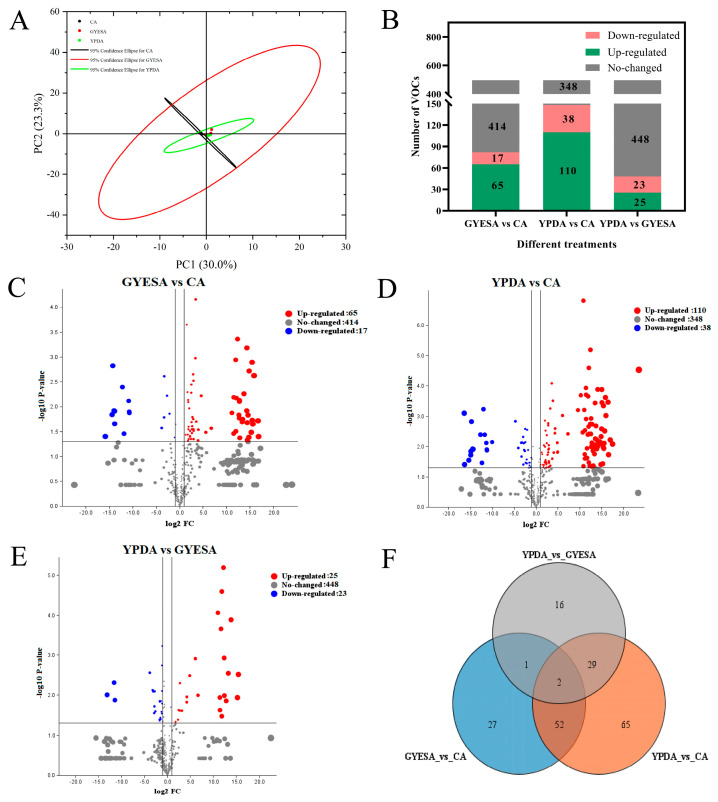
Analysis of total VOCs produced by CEF642^N^ across different media. (**A**) PCA score plot of VOCs. (**B**) Number of up-regulated, down-regulated, and non-significant VOCs in each comparison group. (**C**–**E**) Volcano plots of VOCs in GYESA_vs_CA, YPDA_vs_CA, and YPDA_vs_GYESA groups. (**F**) Venn diagram of differential VOCs across comparison groups.

The VOCs produced by CEF642^N^ mycelium on CA, GYESA, and YPDA media were analyzed using HS-SPME-GC-MS, yielding 593 peaks, of which 496 were annotatable VOCs. Principal component analysis (PCA) revealed that the cumulative contribution of the first two principal components (PC1: 30.0%; PC2: 23.3%) was 52.3%. Biological replicates from the CA and YPDA media formed compact clusters, while those from the GYESA medium were more dispersed (Figure 2A). Differential VOC expression was determined using |log2 FoldChange| > 1 and *p*-value < 0.05 (Figure 2B). The GYESA_vs_CA comparison group exhibited 65 up-regulated, 17 down-regulated, and 414 non-significant VOCs. Similarly, the YPDA_vs_CA group had 110 up-regulated, 38 down-regulated, and 348 non-significant VOCs, while the YPDA_vs_GYESA group showed 25 up-regulated, 23 down-regulated, and 448 non-significant VOCs. Volcano plots (Figure 2C–E) indicated that the GYESA and YPDA media produced more up-regulated VOCs compared to the CA medium, while the GYESA and YPDA media exhibited similar VOC profiles. Venn diagram analysis revealed 52 shared differential VOCs between the GYESA_vs_CA and YPDA_vs_CA groups (Figure 2F).

**Figure 3:**
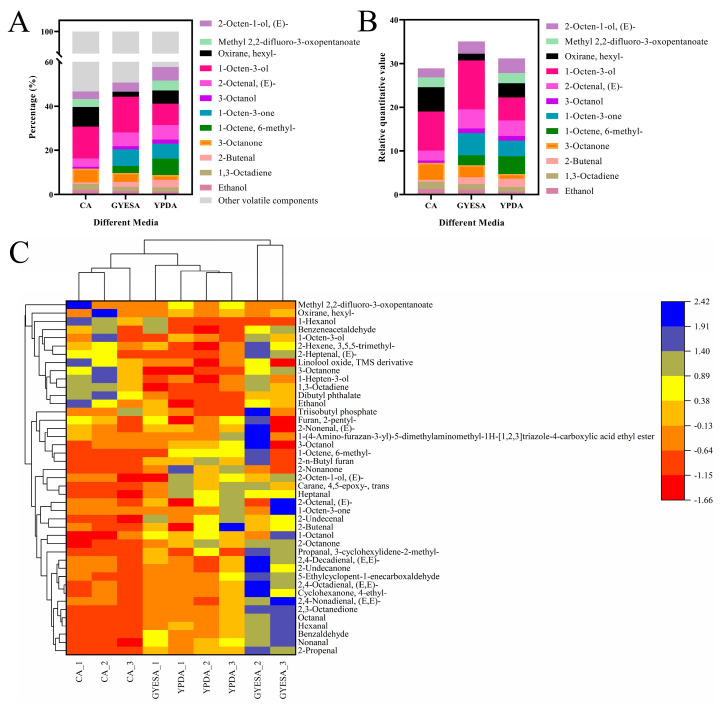
Analysis of major VOCs produced by CEF642^N^ across different media. (**A**) Stacked plot of VOCs percentages. (**B**) Stacked plot of VOCs relative quantitative values. (**C**) Heatmap of major VOCs clustering across media.

A total of 12 annotatable VOCs with relative quantitation values > 1 were identified (Table S2), including ethanol, 1,3-octadiene, 2-butenal, 3-octanone, 1-octen, 6-methyl-, 1-octen-3-one, 3-octanol, 2-octenal, (E)-, 1-octen-3-ol, oxirane, hexyl-, 2-octen-1-ol, (E)-, and methyl 2,2-difluro-3-oxopentanoate. The top three VOCs by percentage and relative quantitation value in each medium was as follows (Figure 3A,B): YPDA medium was 1-octen-3-ol (9.79%; 5.30), 1-octen, 6-methyl- (7.49%; 4.05), and 1-octen-3-one (6.69%; 3.62); GYESA medium was 1-octen-3-ol (16.18%; 11.23), 1-octen-3-one (7.38%; 5.12), and 2-octenal, (E)- (6.26%; 4.34); and CA medium was 1-octen-3-ol (14.41%; 8.93), oxirane, hexyl- (9.02%; 5.59), and 3-octanone (6.28%; 3.89). Clustered heatmap analysis revealed significant differences in the major VOCs among different media (Figure 3C).

**Figure 4:**
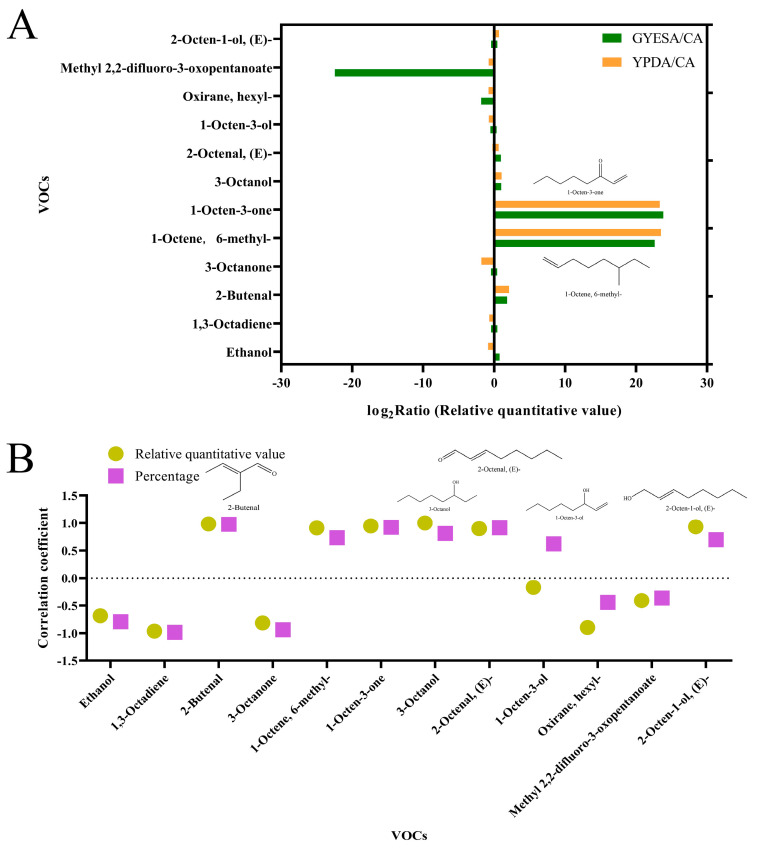
Comparative analysis of primary VOCs and their correlation with inhibition rates. (**A**) Log2-transformed ratios of primary VOCs relative quantitation values in GYESA and YPDA media compared to CA medium. (**B**) Correlation analysis of relative quantitative values and percentages of major VOCs with inhibition rate.

By analyzing the log2-transformed ratios of the mean relative quantitative values of the primary VOCs across the culture media, it was evident that the concentrations of 1-octene, 6-methyl-, and 1-octen-3-one were significantly higher in the GYESA and YPDA media compared to the CA medium (Figure 4A). Furthermore, the VOCs produced by GYESA and YPDA exhibited substantially greater inhibitory effects on Vd076 than those from the CA medium, with inhibition rates of 75.04%, 78.88%, and −4.19%, respectively. A comparative analysis of the major components of the three media, without considering biomass, revealed that the percentage and relative quantitative values of 2-butenal, 1-octene, 6-methyl-, 1-octen-3-one, 3-octanol, 2-octenal, (E)-, and 2-octen-1-ol, (E)- were positively correlated with their respective inhibition rates against *V. dahliae* (Figure 4B). Integrating the percentage and relative quantitative values of the primary components with a comprehensive analysis of inhibition rates, it was hypothesized that the elevated levels of 1-octene, 6-methyl-, and 1-octen-3-one in GYESA and YPDA likely contributed to their higher inhibition rates compared to the CA medium. This hypothesis aligns with previous findings, demonstrating the significantly antifungal activity of 1-octen-3-one against *V. dahliae* [14].

### 3.3. In Vitro Antifungal Activity

**Figure 5:**
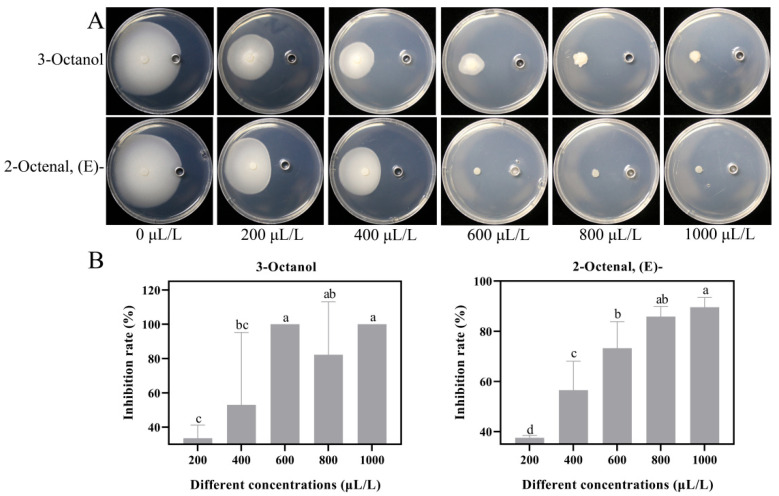
Inhibition of Vd076 by 3-octanol and 2-octen-1-ol, (E)-. (**A**) Photographs of Vd076 treated with varying concentrations of 3-octanol and 2-octen-1-ol, (E)- at day 15. (**B**) Inhibition rates of Vd076 at day 15.

The antifungal activity of total VOCs from the GYESA and YPDA media were significantly higher than that of the CA medium. In addition to previously identified antifungal VOCs (e.g., 3-octanone, 1-octen-3-ol, 2-octen-1-ol, (E)-, and 1-octen-3-one; [14]), standardized samples of 3-octanol and 2-octenal, (E)- were tested. At a concentration of 600 μL/L, 3-octanol and 2-octenal, (E)- inhibited mycelial growth of *V. dahliae* by 73.29% and 100%, respectively (Figure 5), with the median effect concentration (EC_50_) of 493.0 and 404.4 μL/L (Table S3). The EC_50_ values for 3-octanol and 2-octenal, (E)- were higher than those of 3-octanone but lower than 1-octen-3-one, and comparable to 1-octen-3-ol and 2-octen-1-ol, (E)- [14]. Time course revealed that inhibition by 3-octanol increased initially before declining, while inhibition by 2-octenal, (E)- decreased steadily over time (Figure S4).

Both 3-octanol and 2-octenal, (E)- significantly inhibited *V. dahliae* spore germination (Figure S5), with inhibition rates of 72.76% and 53.95% for 3-octanol, and 74.49% and 59.06% for 2-octenal, (E)- at 24 and 48 h, respectively (Figure S6).

### 3.4. Transcriptome Analysis of Mycelium in Different Solid Media

#### 3.4.1. Enrichment Analysis of Fatty Acid Metabolism-Related Genes

**Figure 6:**
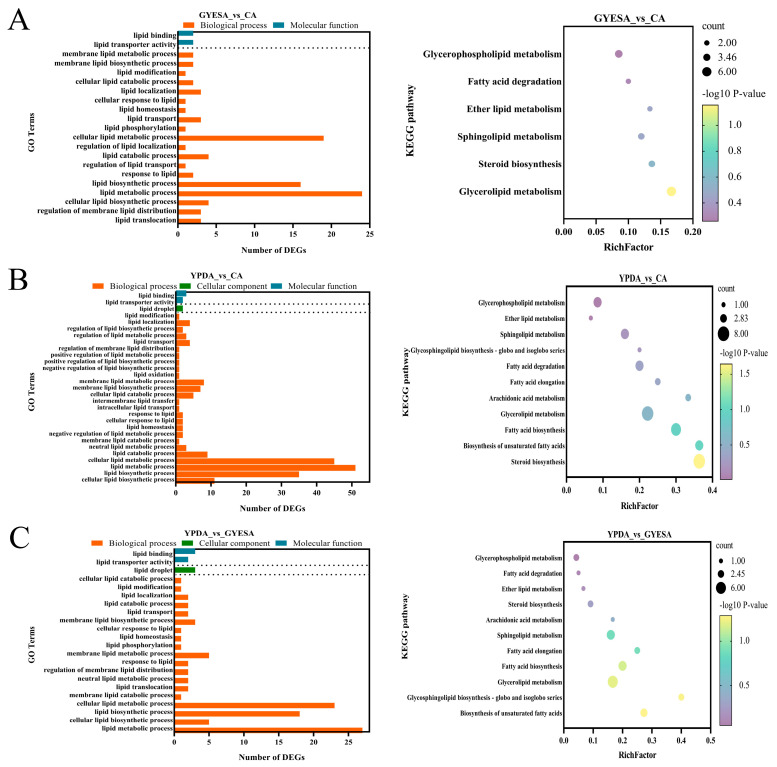
GO and KEGG enrichment analysis of fatty acid metabolism-related genes in CEF642^N^. (**A**–**C**) Enrichment analysis for GYESA_vs_CA, YPDA_vs_CA, and YPDA_vs_GYESA groups.

Fatty acid metabolism-related genes, which are precursors for the biosynthesis of antifungal VOCs such as 1-octen-3-ol [14], were enriched in CEF642^N^. Gene Ontology (GO) enrichment analysis revealed significant involvement in lipid metabolic process and lipid biosynthetic process across all comparison groups. Kyoto Encyclopedia of Genes and Genomes (KEGG) enrichment analysis indicated pathways such as fatty acid degradation (GYESA_vs_CA), biosynthesis of unsaturated fatty acids, fatty acid biosynthesis, arachidonic acid metabolism, and fatty acid degradation (YPDA_vs_CA and YPDA_vs_GYESA) (Figure 6A–C). The expression of genes associated with key differential KEGG enrichment pathways was higher in the YPDA medium compared to the CA medium. This included genes involved in the unsaturated fatty acid biosynthesis pathway *(scaffold6.g636*, *scaffold1.g711*, and *scaffold3.g286*), the fatty acid biosynthetic pathways (*scaffold4.g701*, *scaffold4.702*, *scaffold1.g494*, *scaffold8.g10*, and *scaffold10.g186*), the arachidonic acid metabolic pathways (*scaffold8.g178* and *scaffold1.g597*), and the fatty acid degradation pathway (*scaffold4.g199*, *scaffold1.g1249*, and *scaffold2.g759*) (Figure S7).

#### 3.4.2. Expression of Genes Related to Eight-Carbon VOCs Biosynthesis

**Figure 7:**
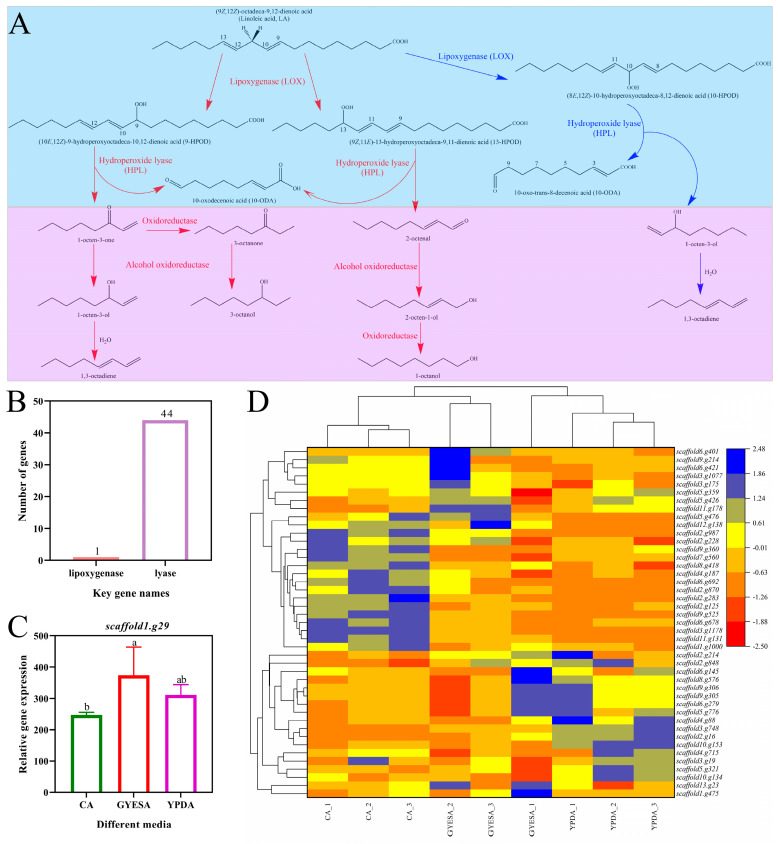
Biosynthetic pathways and gene expression analysis of eight-carbon VOCs in CEF642^N^. (**A**) Proposed pathways for eight-carbon VOCs production [23,24,25,26]. (**B**) Number of LOX and lyase genes in CEF642^N^ genome. (**C**) Expression of LOX gene (*scaffold1.g29*) across media. (**D**) Heatmap of lyase gene expression across media.

Eight-carbon volatiles, which contribute to the distinct scent of many fungi, are oxylipin molecules involved in various biological processes. These compounds are produced through the oxidation and cleavage of linoleic acid [19,20]. Lipoxygenase (LOX) and lipid hydroperoxide lyase (HPL) are the two most important enzymes [21,22] in its biosynthesis (Figure 7A). A genomic search for LOX in the CEF642^N^ genome identified only one gene, *scaffold1.g29*, and no HPL-related genes were detected. However, 44 genes associated with lyase enzymes were identified (Figure 7B). Among the three different media, the LOX gene *scaffold1.g29* exhibited the highest expression in the GYESA medium, followed by the YPDA medium (Figure 7C). Lyase enzyme genes, including *scaffold2.g848*, *scaffold3.g19*, *scaffold4.g715*, *scaffold10.g134*, *scaffold5.g321*, *scaffold5.g359*, *scaffold5.g426*, *scaffold3.g748*, *scaffold10.g153*, *scaffold2.g16*, *scaffold4.g88*, *scaffold9.g305*, *scaffold9.g306*, *scaffold5.g776*, *scaffold6.g279*, and *scaffold8.g576*, showed higher expression in the YPDA medium compared to the CA medium (Figure 7D).

## 4. Discussion

Agricultural soils are commonly treated with pesticides to control nematodes, soil-borne pathogens, and weeds, particularly in preparation for planting high-value cash crops. Ideally, pesticide should selectively target harmful organisms; however, fumigants, a class of pesticides with broad biocidal activity, often affect many non-target soil organisms [27]. Plant endophytic fungi produce VOCs that hold significant potential for biological control due to their small molecular size and ease of dispersal in plants and soils. These fungal VOCs offer an environmentally benign, cost-effective, and sustainable solution for agricultural practices [28].

The inhibitory effect of VOCs produced by *T. purpureogenus* CEF642^N^ on *V. dahliae* varied across three different media: minimal inhibitory activity was observed on the CA medium, while significant inhibition occurred on the GYESA and YPDA media (Figure 1). This suggests that VOC production by CEF642^N^ is media-dependent. To investigate this further, we conducted a comparative metabolomic analysis of VOCs across the three media (Figure 2 and Figure 3). The results revealed that 1-octen-3-one and 1-octene, 6-methyl- were present in significantly higher concentrations in the GYESA and YPDA media compared to the CA medium (Figure 4). Notably, 1-octen-3-one demonstrated strong antagonistic activity against *V. dahliae* mycelium growth [14], which may explain the higher rates observed in the GYESA and YPDA media. Additionally, 3-octanol and 2-octenal, (E)- were identified as major VOCs in the GYESA and YPDA media, accounting for 1.49%, 2.00% (3-octanol) and 6.26%, 6.49% (2-octenal, (E)-), respectively. These compounds significantly inhibited mycelial growth and spore germination of *V. dahliae* (Figure 5 and Figure S6). 3-Octenal, a safe fungal VOC, was reported to inhibit *Botrytis cinerea* by inducing autophagy, reducing cell viability, and suppressing spore germination [29]. Similarly, *trans*-2-octenal derived from *Lactiplantibacillus plantarum* exhibits antifungal activity against *Aspergillus niger* CECT 2805 [30]. In addition, *Bacillus velezensis* SEC-024A showed strong antifungal effects against industrial hemp wilt, with VOCs identified as the main antifungal components. Among these, *trans*-2-octenal demonstrated broad-spectrum activity against soil-borne pathogens [31]. Furthermore, (*E*)-2-octenal inhibits the growth of postharvest pathogens (*Neofusicoccum parvum* and *Penicillium italicum*) by disrupting mitochondrial energy metabolism, suggesting its potential as a postharvest preservative for citrus and mango [32,33]. To our knowledge, this is the first report of significant antagonistic activity of 3-octanol and 2-octenal, (E)- against *V. dahliae*. These findings provide a theoretical foundation for developing biofumigants to control cotton Verticillium wilt.

Certain enzymes and SMs are uniquely synthesized through solid-state fermentation (SSF) owing to the distinctive physiological micro-environment established in this cultivation system. Higher yields of SMs are associated with increased transcription of biosynthetic genes, and studies on SSF enzyme production have identified SSF-specific genes, offering insights into their expression and regulation [34]. Fatty acids serve as precursors for eight-carbon VOCs, which exhibit significant antagonistic effect [35]. KEGG enrichment analyses of transcriptome data revealed that differentially expressed genes are primarily involved in unsaturated fatty acid biosynthesis and arachidonic acid metabolism, among other things (Figure 6). Arachidonic acid is metabolized via two pathways: the cyclooxygenase (COX) and LOX pathways [36]. Research on *Marchantia polymorpha* has shown that 1-octen-3-ol, the primary C8 product of arachidonic acid metabolism, undergoes further modification, including acetylation and redox reaction, to diversify C8 compounds [37].

Although eight-carbon VOCs play crucial roles in many biological activities, their biosynthetic pathways in fungi remain poorly understood. The synthesis of 1-octen-3-ol is relatively well documented, though some hypotheses lack sufficient evidence. Fungi utilize fatty acids to produce volatile compounds, which are subsequently oxidized and cleaved into short-chain VOCs. LOX, a key enzyme in oxylipin synthesis, catalyzes the initial step in the conversion of polyunsaturated fatty acids to oxylipins [38,39]. Its activity and transcriptional regulation are critical for oxylipin synthesis [40,41]. HPL, an enzyme downstream of LOX, cleaves LOX products into short-chain aldehydes and oxygenated acids [42,43]. For instance, adding linoleic acid to the culture medium enhanced 1-octen-3-ol production by *Penicillium camemberti*, particularly through increased HPL activity [44]. Similarly, in *Agaricus bisporus*, LOX and HPL are essential for 1-octen-3-ol synthesis from linoleic acid [45], with *Ab*LOX and *Ab*HPL identified as the key enzymes [46]. Shiitake mushrooms cultivated on maize cobs produced the highest levels of eight-carbon VOCs [47]. Transcriptomics analysis of selected genes, combined with VOC profiling at different developmental phases, has proven effective in identifying enzymes involved in fungal VOC biosynthesis [48]. In this study, the analysis of differential expression patterns of key genes involved in the eight-carbon VOCs biosynthesis pathway in CEF642^N^ under different culture media conditions identified critical targets for further screening to enhance 1-octen-3-ol production.

## 5. Conclusions

This study employed the OSMAC strategy to investigate the antagonistic activity of VOCs produced by *T. purpureogenus* CEF642^N^ against *V. dahliae* across three media. Comparative metabolomic analysis revealed differences in VOC composition, particularly the higher proportion of 1-octen-3-one in the GYESA and YPDA media, which likely explains the observed differences in the inhibition rates. The primary antifungal VOCs, 3-octanol and 2-octenal, (E)-, demonstrated significant inhibitory effects on *V. dahliae* mycelial growth and spore germination, highlighting their potential as green pesticides or biofumigants for controlling cotton Verticillium wilt. Transcriptome analysis of mycelia grown on different media identified differentially expressed genes involved in lipid metabolism and eight-carbon VOCs biosynthesis, providing a theoretical basis for future gene screening and biosynthesis studies.

## Data Availability

The original contributions presented in this study are included in the article/Supplementary Materials. Further inquiries can be directed to the corresponding authors.

## Supplementary Materials

The following supporting information can be downloaded at: https://www.mdpi.com/article/10.3390/jof11050332/s1. Table S1: Composition of three solid culture media; Table S2: Analysis of major VOCs (relative quantitative value > 1) produced by *T. purpureogenus* CEF642^N^ in different culture media; Table S3: EC_50_ value of 3-octanol and 2-octenal, (E)- for inhibiting mycelial development of *V. dahliae*; Figure S1: Time course of double Petri dish assay between CEF642^N^ and Vd076 on CA medium; Figure S2: Time course of double Petri dish assay between CEF642^N^ and Vd076 on GYESA medium; Figure S3: Time course of double Petri dish assay between CEF642^N^ and Vd076 on YPDA medium; Figure S4: Inhibition rates of *V. dahliae* mycelial growth at different time points under varying concentrations of 3-octanol and 2-octenal, (E)-; Figure S5: Images of *V. dahliae* spore germination after treatment with 1 μL of 3-octanol and 2-octenal, (E)-; Figure S6: Inhibition rate of *V. dahliae* spore germination following treatment with 1 μL of 3-octanol and 2-octenal, (E)-; Figure S7: Heatmap on differential gene expression in fatty acid metabolic pathway from KEGG enrichment analysis of different solid media.

## Abbreviations

The following abbreviations are used in this manuscript:

VOCs	Volatile organic compounds
OSMAC	One strain many compounds
LOX	Lipoxygenase
SMs	Secondary metabolites
HS-SPME	Headspace solid-phase microextraction
PAL	Prep And Load
GC-MS	Gas chromatography–mass spectrometry
CA	Czapek agar
GYESA	Glucose yeast extract soluble starch agar
YPDA	Yeast extract peptone dextrose agar
PDA	Potato dextrose agar
LSD	Least significant difference
ANOVA	Analysis of variance
SD	Standard deviation
PCA	Principal component analysis
EC_50_	Median effect concentration
GO	Gene Ontology
KEGG	Kyoto Encyclopedia of Genes and Genomes
HPL	Hydroperoxide lyase
SSF	Solid-state fermentation
