# Supplementary material for: Antifungal Volatile Organic Compounds from Talaromyces purpureogenus CEF642N: Insights from One Strain Many Compounds (OSMAC) Strategy for Controlling Verticillium dahliae in Cotton
Source: J Fungi (Basel). 2025 Apr 22;11(5):332. doi: 10.3390/jof11050332 (PMC12112887; doi:10.3390/jof11050332)
Supplement: Supplementary file 1 [file jof-11-00332-s001.zip › jof-3550576-supplementary.pdf]

Supplementary Materials:

Antifungal Volatile Organic Compounds from *Talaromyces purpureogenus* CEF642[N]: Insights from One Strain Many Compounds (OSMAC) Strategy for Controlling *Verticillium dahliae* in Cotton

Peng Li [1,†], Yalin Zhang [1,2,†], Hongjie Feng [1,2], Jinglong Zhou [1,2], Lihong Zhao [1,2], Heqin Zhu [1,2], Feng Wei [1,2,*] and Zili Feng [1,2,*]

Table S1. Composition of three solid culture media.

Culture media	Main components (per Liter)
Czapek Agar (CA)	$NaNO_3$ 2.0 g, KCl 0.5 g, $FeSO_4 \cdot 7H_2O$ 0.02 g, $MgSO_4 \cdot 7H_2O$ 0.5 g, sucrose 30 g, K_2HPO_4 1.31 g, agar 15 g
Glucose Yeast Extract soluble Starch Agar (GYESA)	Glucose 10 g, soluble starch 10 g, yeast extract 10 g, NaCl 5 g, $CaCO_3$ 3 g, agar 15 g
Yeast extract Peptone Dextrose Agar (YPDA)	Glucose 20 g, peptone 20 g, yeast extract 10 g, agar 15 g

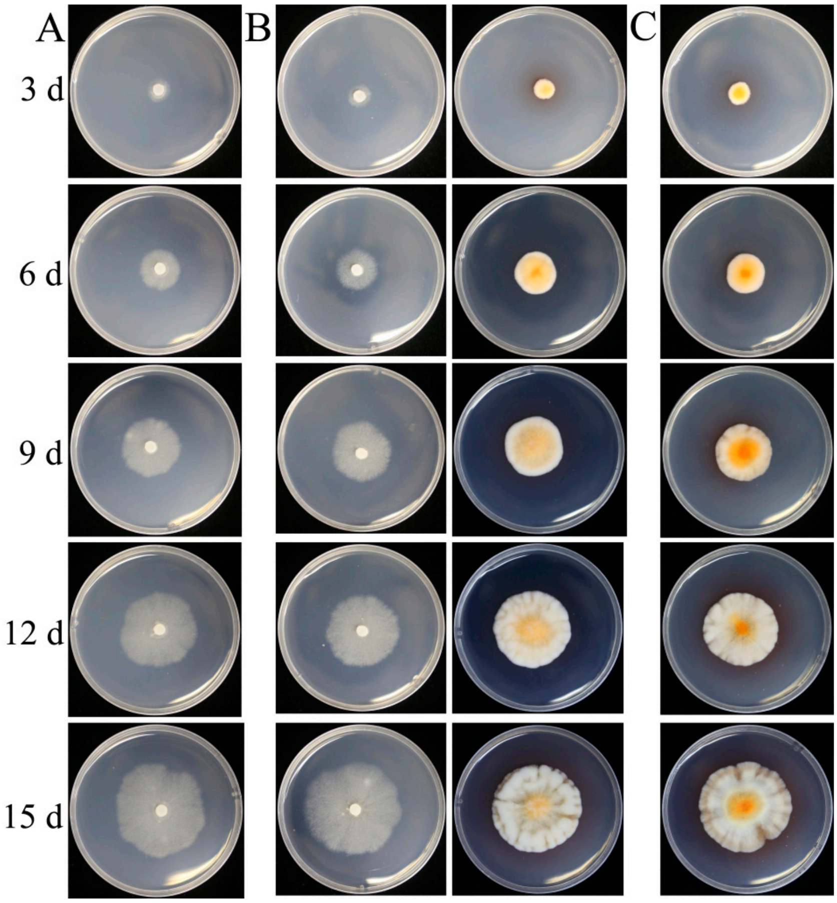

Figure S1. Time course of double Petri dish assay between CEF642^{N} and Vd076 on CA medium. (A) Vd076 grown alone. (B) CEF642^{N} (right) and Vd076 (left) co-cultured in double Petri dish assay. (C) CEF642^{N} grown alone.

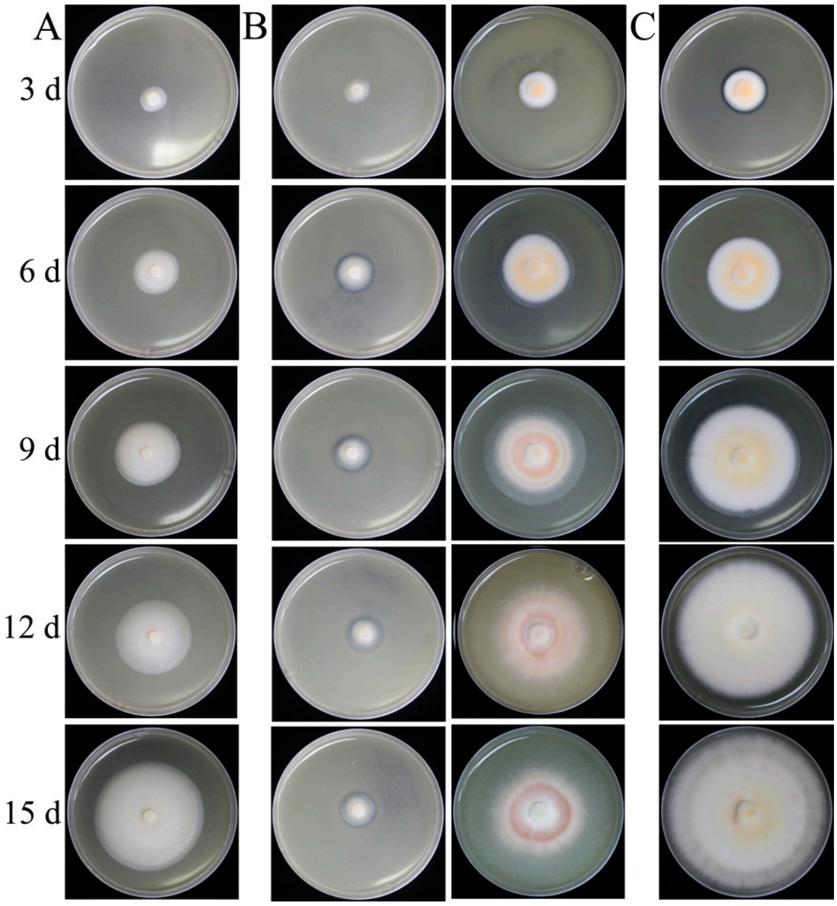

Figure S2. Time course of double Petri dish assay between CEF642^{N} and Vd076 on GYESA medium. (A) Vd076 grown alone. (B) CEF642^{N} (right) and Vd076 (left) co-cultured in double Petri dish assay. (C) CEF642^{N} grown alone.

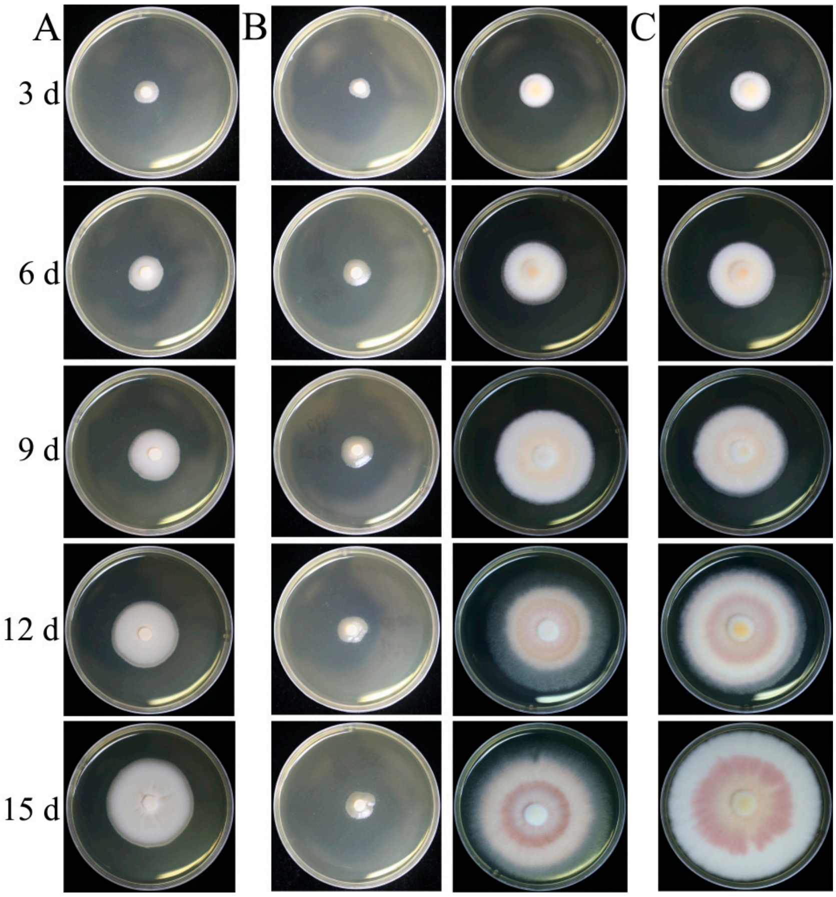

Figure S3. Time course of double Petri dish assay between CEF642N and Vd076 on YPDA medium. (A) Vd076 grown alone. (B) CEF642N (right) and Vd076 (left) co-cultured in double Petri dish assay. (C) CEF642^{N} grown alone.

Table S2. Analysis of major VOCs (relative quantitative value > 1) produced by *T. purpureogenus* CEF642^{N} in different culture media.

Retention time (min)	Compound name	Similarity	MW	Molecular formula	Mean			CAS
					CA	GYESA	YPDA	
3.701	Ethanol	917	46.068	C_2H_6O	1.268613142	1.155135356	0.701957488	64-17-5

4.012	1,3-Octadiene	906	110.197	C_8H_{14}	1.678299911	1.244706418	1.042697703	1002-33-1
5.901	2-Butenal	928	70.090	C_4H_6O	0.437820205	1.537986486	1.857348801	4170-30-3
12.228	3-Octanone	891	128.212	$C_8H_{16}O$	3.889057986	2.826730237	1.107097818	106-68-3
13.732	1-Octene, 6-methyl-	739	126.239	C_9H_{18}	3.40934E-07	2.222552183	4.051702804	-
13.768	1-Octen-3-one	774	126.196	$C_8H_{14}O$	3.40934E-07	5.120334667	3.619198146	4312-99-6
16.349	3-Octanol	885	130.228	$C_8H_{18}O$	0.523956027	1.036657857	1.080024756	70492-66-9
17.096	2-Octenal, (E)-	867	126.196	$C_8H_{14}O$	2.276083077	4.341242464	3.508985156	2548-87-0
17.925	1-Octen-3-ol	767	128.212	$C_8H_{16}O$	8.927237145	11.22922656	5.295484477	3391-86-4
17.977	Oxirane, hexyl-	715	128.212	$C_8H_{16}O$	5.586215406	1.556010804	3.208387954	77495-66-0
21.931	2-Octen-1-ol, (*E*)-	866	128.212	$C_8H_{16}O$	2.099480973	2.836021905	3.338893801	18409-17-

21.934	Methyl<br>2,2-difluoro<br>-3-oxopenta<br>noate	625	166.<br>123	$C_6H_8F_2O_3$	2.2164<br>85479	3.91095<br>E-07	2.3832<br>143	1<br>1962<br>02-01<br>-4

Table S3. EC_{50} value of 3-octanol and 2-octenal, (E)- for inhibiting mycelial development of *V. dahliae*.

VOCs	EC_{50} (μL/L)
3-Octanol	493.0
2-Octenal, (E)-	404.4

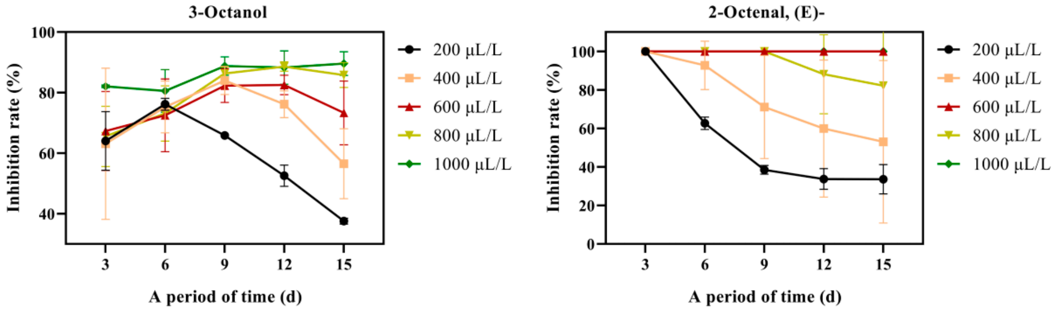

Figure S4. Inhibition rates of *V. dahliae* mycelial growth at different time points under varying concentrations of 3-octanol and 2-octenal, (E)-.

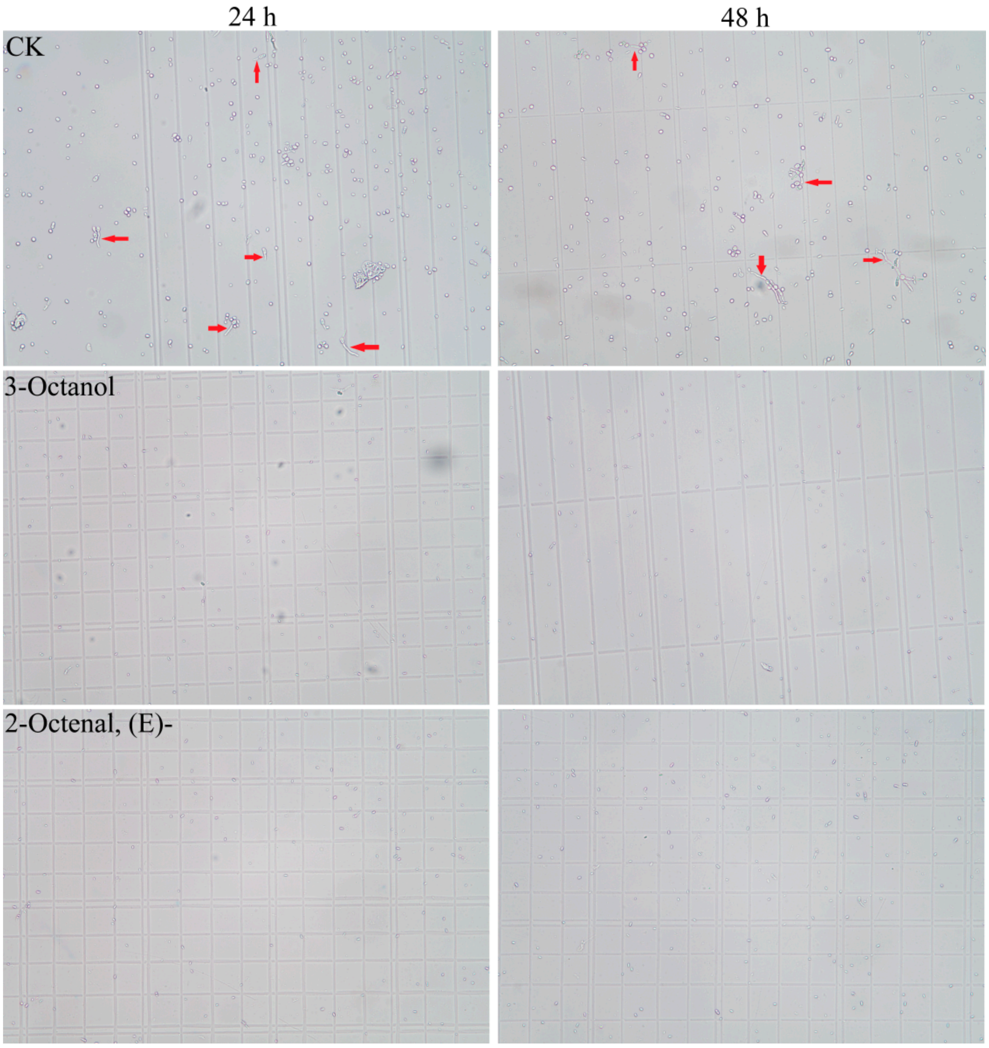

Figure S5. Images of *V. dahliae* spore germination after treatment with 1 μL of 3-octanol and 2-octenal, (E)-.

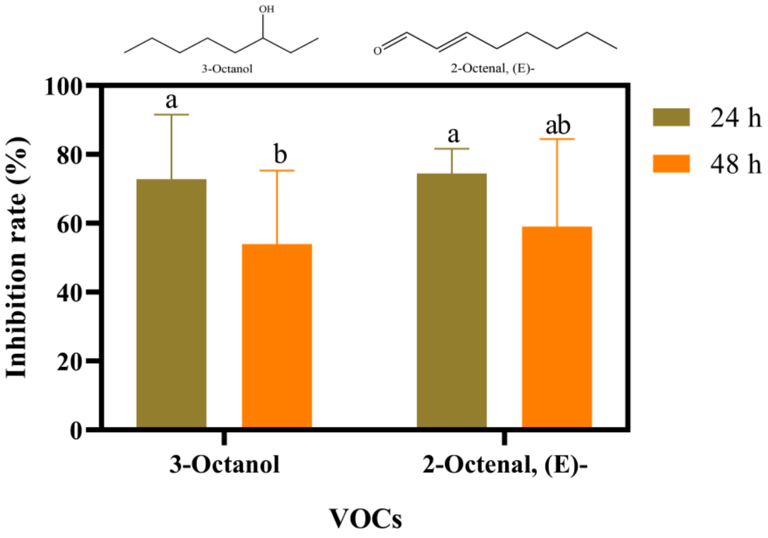

Figure S6. Inhibition rate of *V. dahliae* spore germination following treatment with 1 μL of 3-octanol and 2-octenal, (E)-.

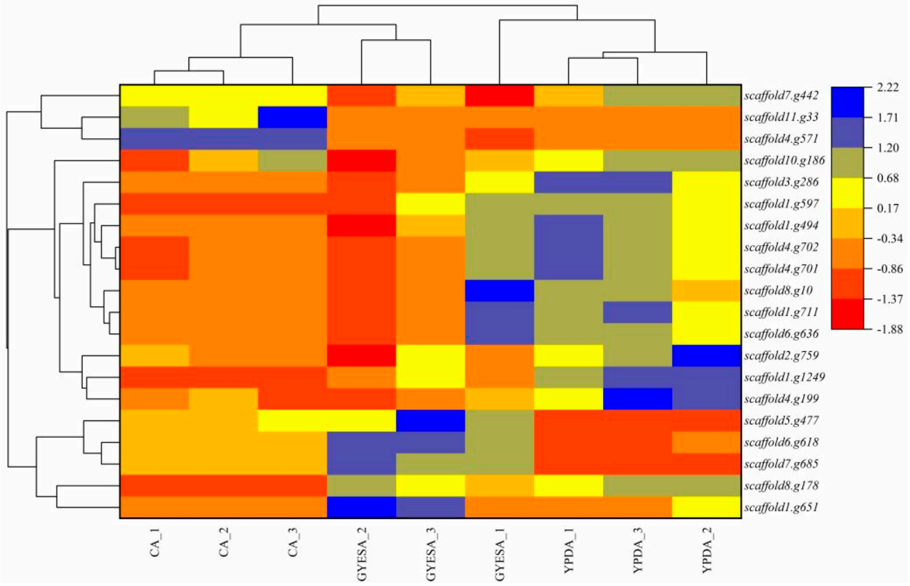

Figure S7. Heatmap on differential gene expression in fatty acid metabolic pathway from KEGG enrichment analysis of different solid media.
